# Early Rising

**Published:** 1879-07

**Authors:** 


### EARLY RISING.

A FRIEND has sent us *The Weekly Comet*, of Baton Rouge, Louisiana, whose editor gives us a column of epithets for "coming out in our book against early rising," and in the high excitement of the subject exclaims—"Holy Moses and the lamb, is there no truth?" This is one of the many cases where a man sits down to criticise what he never read. So far from opposing early rising, we have recommended it in all our writings; always and everywhere, by published theory and private practice, advocating the doctrine that "*it was one of the safest, wisest, healthiest, and most profitable practices, to go to bed early, get up early, eat breakfast and go to work.*" If any one can fabricate a more sensible platform than that we would like to make his acquaintance.

Either we or some of our readers are decidedly obfuscated.

*The Water-Cure Journal*, in reply to a correspondent inquiring if "Dr. HALL'S theory against early rising was a true one," answers—"Fudge"; which is so conclusive an answer that we have nothing more to add.

Another editor, who also imbibed the idea that we asserted that early rising was unhealthful, exclaims in the exuberance of his intolerance—"Do not the birds get up by daylight, and are they not the most healthy people in the world?" Why yes, certainly. How could you imagine that we thought them unhealthy? The argument is unanswerable. How few of us read and think! How many skim!

